# Retrospective comparative analysis of pre-IVF vaginal and semen microbiological cultures in infertile couples highlights absence of microbial concordance

**DOI:** 10.3389/fcimb.2026.1747088

**Published:** 2026-04-29

**Authors:** Anna Vágvölgyi, Bernadett Nádasdi, Petra Lilla Zádori, Rita Sinka, Katalin Burián, Viktor Vedelek, János Zádori

**Affiliations:** 1 Department of Medicine, Albert Szent-Györgyi Medical School, University of Szeged, Szeged, Hungary; 2 Center of Reproductive Medicine, Department of Obstetrics and Gynecology, Albert Szent-Györgyi Medical School, University of Szeged, Szeged, Hungary; 3 Department of Genetics, Faculty of Science and Informatics, University of Szeged, Szeged, Hungary; 4 Department of Medical Microbiology, Albert Szent-Györgyi Health Center and Albert Szent-Györgyi Medical School, University of Szeged, Szeged, Hungary

**Keywords:** clinical pregnancy, *in vitro* fertilization, lactobacilli colonization, reproductive tract infections, vaginal and semen microbiological culture

## Abstract

**Introduction:**

Infertility affects 15–20% of couples, with 3% of Hungarian children conceived through *in vitro* fertilization (IVF). Predicting IVF outcomes before treatment initiation remains challenging. Emerging evidence suggests that the vaginal and seminal microbiota influence reproductive health by modulating local immunity, implantation, and gamete function. This study aimed to evaluate associations between baseline clinical, biochemical, and microbiological parameters and IVF outcomes.

**Material and methods:**

We conducted a retrospective, single-center cohort study including 475 couples undergoing IVF with or without intracytoplasmic sperm injection at the University of Szeged (January 2022–December 2023). Data collection encompassed maternal demographics, reproductive history, baseline hormone levels, ovarian stimulation characteristics, and endometrial thickness. The results of microbiological cultures of vaginal discharge and semen samples, including *Lactobacillus* colonization, pathogen distribution, and the antibiotic resistance status of the pathogens, were recorded. The primary outcome was clinical pregnancy. Machine learning models, including support vector machine (SVM), random forest (RF), and extreme gradient boosting (XGBoost) were applied, to explore the predictive value of combined clinical and microbial features for IVF outcome.

**Results:**

Positive vaginal cultures were identified in 121 women (25.5%), most commonly *Candida albicans* (26%), *Streptococcus agalactiae* (17%), and *Escherichia coli* (17%) among the positive cases. Among 134 men (29%) with positive semen cultures, *Enterococcus faecalis* (46%) and *Escherichia coli* (23%) predominated. Single-organism growth predominated, and pathogen overlap between partners was rare (13 couples, <3%). Vaginal *Lactobacillus* presence negatively correlated with several pathogens, including *Candida albicans*, *Streptococcus agalactiae*, *Gardnerella vaginalis*, and *Enterococcus faecalis*. Clinical pregnancy rates were similar between women with positive and negative vaginal cultures (36% vs. 39%, n.s.). Machine learning analyses showed that maternal age remained the dominant predictor, while microbial data contributed modestly, with *Lactobacillus* consistently emerging as the most relevant microbial feature.

**Conclusions:**

Vaginal and seminal microbial alterations are common among couples undergoing IVF, yet true pathogen overlap between partners was minimal. *Lactobacillus* colonization demonstrated a clear protective association, supporting its potential as a biomarker for reproductive success. Although microbial features alone are insufficient to predict IVF outcomes, integrating microbial profiles with established clinical parameters could inform personalized fertility management.

## Introduction

Infertility itself affects approximately 15–20% of couples (EMMI, 2019), and in Hungary about 3% of children are conceived through assisted reproductive technologies. *In vitro* fertilization (IVF) has fundamentally reshaped reproductive medicine, providing a realistic chance of parenthood for couples and individuals experiencing infertility. The variability in treatment results has driven extensive research on reliable indicators of treatment success before initiation—described as preprocedural factors—has become a major focus of current scientific investigation (Bereczki et al., 2025; Nádasdi et al., 2025). Accordingly, baseline clinical history, biometric characteristics, initial laboratory measures, as well as microbiological cultures of vaginal secretions and semen are increasingly being evaluated as potential determinants of outcome. Vaginal microbial communities can greatly influence maternal health and have important effects on fetal and neonatal outcomes (Hillier et al., 1995; Krohn et al., 1997). Normally, the vaginal microbiota of women is dominated by *Lactobacillus species* (Chenoll et al., 2019; Tomaiuolo et al., 2020; Mohammadi et al., 2025). *Lactobacilli* prevent colonization by harmful microbes; however, their depletion creates an opportunity for bacteria such as *Streptococcus* and *Gardnerella* to expand (Chenoll et al., 2019). Alterations in the vaginal microbiota have been linked to a considerable decrease in pregnancy success rates after IVF (Hyman et al., 2012; Haahr et al., 2016; Tomaiuolo et al., 2020). Clinical pregnancies were observed in only a small proportion of women with vaginal dysbiosis, characterized by elevated levels of *Gardnerella vaginalis* and/or *Atopobium vaginae*, suggesting that such microbial imbalances may have a detrimental effect on IVF outcomes (Haahr et al., 2016). Alterations in the microbial environment can contribute to infertility by causing direct damage to the reproductive tract, disrupting implantation and fertilization processes, and decreasing both the motility and viability of sperm (Zhang et al., 2021). The male reproductive tract microbiota may also influence IVF outcomes, with alterations in bacterial composition potentially contributing to seminal abnormalities such as increased viscosity and impaired sperm parameters. These changes have been associated with an overrepresentation of potentially pathogenic species and a reduction in beneficial commensal bacteria (Monteiro et al., 2018). The primary objective of this retrospective study was to investigate potential associations between baseline clinical and biochemical parameters, and vaginal and seminal microbiological analysis outcomes. A key focus was to determine whether women exhibiting a balanced vaginal microbiota, characterized by the presence of lactic acid–producing bacteria, demonstrated a lower prevalence of infections within their couples compared to those with dysbiotic vaginal flora.

Additionally, the study evaluated correlations between pathogen presence, baseline lactobacilli colonization, and various reproductive parameters, including clinical pregnancy rates, alongside patient demographics and hormonal profiles.

An analysis of concordance between positive microbiological culture results in female and male partners was also performed, with particular attention to the proportion of cases exhibiting identical pathogens versus differing microbial profiles within couples.

## Materials and methods

### Study population

A retrospective, single-center cohort study was conducted at the Center of Reproductive Medicine, Department of Obstetrics and Gynecology, Albert Szent-Györgyi Medical School, University of Szeged in cooperation with the Department of Medical Microbiology, University of Szeged, Szeged, Hungary. The data collection was performed from January 1, 2022, to December 31, 2023 among women treated for IVF with or without intracytoplasmic sperm injection (ICSI) with successful oocyte retrieval.

For each participant, biometric characteristics were recorded, including maternal age, body weight, height, and body mass index (BMI; kg/m^2^). Preprocedural information encompassed the duration of infertility (years), reproductive history such as previous live births (n; %), miscarriages (n; %), and induced abortions (n; %), as well as baseline serum levels of follicle-stimulating hormone (FSH; IU/L), luteinizing hormone (LH; IU/L), and anti-Müllerian hormone (AMH; pmol/L). Microbiological and microscopic assessments of the vaginal samples were also performed, comprising the detection of Lactobacillus in culture, microscopic evidence of bacterial vaginosis, and the presence of granulocytes. Procedural data were collected regarding the duration of ovarian stimulation (days), the number of developing follicles, the day and number of embryo transfers, and serial measurements of endometrial thickness at the time of ovulation trigger (ENDOV; mm), follicular puncture (ENDPU; mm), and embryo transfer (ENDET; mm). On the paternal side, age, results of semen microbiological cultures, and semen quality parameters—including concentration (x 10^6^/mL), motility (%), and the proportion of morphologically normal spermatozoa (n; %) — were assessed.

The primary outcome measure was the occurrence of clinical pregnancy. Clinical pregnancy was defined at a gestational age of 7 weeks, as recommended by the International Committee for Monitoring Assisted Reproductive Technology (ICMART) and the World Health Organization (WHO) (Zegers-Hochschild et al., 2009), by the visualization of one or more gestational sacs (including ectopic pregnancies) using transvaginal ultrasound. Biochemical pregnancy, clinical miscarriage, and ectopic pregnancy (also defined by ICMART and the WHO) (Zegers-Hochschild et al., 2009) were recorded separately, and patients with these outcomes were not included in the pregnant group.

Inclusion required the availability of microbiological culture results of vaginal discharge and/or semen. During the aforementioned time interval, complete microbiological culture data of vaginal discharge and semen were available for 475 couples undergoing *in vitro* fertilization and embryo transfer treatment at the Center of Reproductive Medicine, Department of Obstetrics and Gynecology, Albert Szent-Györgyi Medical School, University of Szeged.

### Methods

Ovarian stimulation was performed using individualized GnRH agonist or antagonist protocols selected according to ovarian reserve, hormonal status, age, and body weight. Cycles commenced on day 2–3 with baseline evaluation by transvaginal ultrasound (Samsung Medison HS50; EVN4–9 probe, 4–9 MHz), antral follicle count, and serum FSH, LH, prolactin, and TSH measurement. All laboratory and embryo transfer procedures followed uniform, previously published methods (Vedelek et al., 2024) (**Supplementary Material**). Endometrial thickness was measured on three occasions with the same ultrasound unit: at human chorionic gonadotropin (hCG) administration (ENDOV), at oocyte retrieval (ENDPU), and at embryo transfer (ENDET). Thickness was defined as the maximal distance between the outer margins of the endometrium across the myometrial interface (Sakamoto, 1985).

The results of microbiological culture and microscopic analysis were obtained from multiple diagnostic centers, located in several cities across three countries (Hungary, Romania, and Serbia). This reflects the real-world data nature of our study. The non-uniform methodology was acknowledged as a limitation and is discussed in the corresponding section. When the microbiological analysis report did not identify a specific species and only provided a genus-level designation (spp.), this designation was used in the dataset.

Several yeast species that were formerly classified within the *Candida* genus have undergone taxonomic reclassification based on phylogenetic analyses. Specifically, *Candida krusei* is now recognized as *Pichia kudriavzevii*, *Candida glabrata* as *Nakaseomyces glabrata*, and *Candida guilliermondii* as *Meyerozyma guilliermondii*. As these species were traditionally included among *Candida* spp., their occurrences are discussed within that group for consistency and comparability with earlier studies.

### Microbiological culture and identification

Semen/ejaculate and vaginal specimens were inoculated onto blood agar (bioMérieux, Marcy-l’Étoile, France), chocolate agar (bioMérieux), eosin–methylene blue agar (Oxoid, Basingstoke, UK), and Sabouraud dextrose agar (Oxoid), as well as onto blood agar plates intended for anaerobic incubation (bioMérieux). Before the inoculation, smears were prepared from the samples on sterile glass slides and stained with methylene blue and Gram stain (Merck, Darmstadt, Germany) to assess the presence of leukocytes and to evaluate bacterial vaginosis and the presence of *Neisseria gonorrhoeae*. Following microscopic examination, vaginal samples showing cells characteristic of bacterial vaginosis and lacking normal *Lactobacillus* flora were additionally inoculated onto blood agar plates and incubated under anaerobic conditions. Samples containing numerous leukocytes were also inoculated onto Thayer–Martin selective agar (bioMérieux) for the detection of *N. gonorrhoeae*. Blood agar and chocolate agar plates were incubated for 16–24 h at 35 °C in a 5% CO_2_ atmosphere. Thayer–Martin agar plates were incubated for up to 72 h at 35 °C in a CO_2_ incubator. Eosin–methylene blue agar and Sabouraud agar plates were incubated for 16–24 h at 35 °C. Blood agar plates used for anaerobic culture were incubated for at least 5 days at 37 °C under anaerobic conditions. Sabouraud agar plates were further incubated for an additional 6 days at room temperature. If no growth was observed after 18–24 h of incubation, the incubation time of the plates was extended to 72 h. Detection of *Ureaplasma* spp. was performed using Mycoplasma/Ureaplasma selective broth and agar media (Mycoplasma IST2 system, bioMérieux) according to the manufacturer’s instructions. Inoculated media were incubated at 35–37 °C under appropriate atmospheric conditions and examined for characteristic growth and color change associated with urease activity.

Presumptive isolates were identified using the MALDI Biotyper system (Bruker Daltonik GmbH, Bremen, Germany) or by conventional microbiological methods including colony morphology, biochemical tests, and automated identification using the VITEK 2 system (bioMérieux) according to the manufacturer’s instructions. MALDI-TOF MS identification was accepted at the species level when the log(score) value was ≥2.0. Colony growth was evaluated qualitatively or semi-quantitatively based on colony density on agar plates. Because swab samples do not allow precise volume standardization, colony counts (CFU determination) were not performed.

### Screening for *Streptococcus agalactiae*

Screening for *S. agalactiae* in women was performed in accordance with the CDC 2010 recommendations (Verani et al., 2010). Clinical samples were directly inoculated onto Columbia agar supplemented with 5% sheep blood (bioMérieux) and incubated at 35–37 °C in a 5% CO_2_ atmosphere. In parallel, selective enrichment was performed using modified Todd–Hewitt broth (Oxoid) supplemented with nalidixic acid (0.015 g/L) and colistin (0.010 g/L) and incubated under the same conditions. After 18–24 h of incubation, the enriched broth cultures were subcultured onto CHROMagar StrepB plates (CHROMagar, Paris, France) and incubated aerobically at 37 °C for 18–48 h. Following incubation, Columbia blood agar and CHROMagar StrepB plates were examined for colonies suggestive of *S. agalactiae*. Presumptive isolates were identified using the MALDI Biotyper system (Bruker Daltonik GmbH) or by conventional microbiological methods including colony morphology, β-hemolysis, bacitracin resistance, CAMP reaction, and confirmation of Lancefield group B antigen using a latex agglutination test (Pastorex Strep Kit, Bio-Rad, Marnes-la-Coquette, France). MALDI-TOF MS identification was accepted at the species level when the log(score) value was ≥2.0.

### Antimicrobial susceptibility testing

Antimicrobial susceptibility testing of clinically relevant aerobic and facultative anaerobic bacterial isolates was performed using the automated VITEK 2 system (bioMérieux) according to the manufacturer’s instructions. The results were interpreted in accordance with the clinical breakpoints defined by the European Committee on Antimicrobial Susceptibility Testing (EUCAST). For selected isolates, susceptibility testing was additionally confirmed using the disk diffusion method on Mueller–Hinton agar plates (Oxoid) supplemented with 5% sheep blood (bioMérieux), depending on the bacterial species and EUCAST recommendations. After incubation at 35 ± 1 °C for 18–24 h, inhibition zone diameters were measured and interpreted according to EUCAST breakpoint tables. Antibiotics included in the susceptibility testing panels were selected according to the bacterial species and clinical relevance. Antimicrobial susceptibility testing of *Ureaplasma* spp. was performed using the Mycoplasma IST2 system (bioMérieux) according to the manufacturer’s instructions.

### Statistical analysis

For general data handling, we utilized Microsoft Excel. For data analyses and discovery, the Jupyter notebook v. 6.3.0. was used with Python 3.6. The NumPy (1.22.4), pandas (1.2.4), scikit-learn (1.3.2), imblearn (0.12.4), xgboost (2.1.3), shap (0.44.1), and skopt (0.10.2) libraries were applied for data management, statistical analyses, and model training. For normally distributed continuous variables, Welch’s two-tailed t-test was used to determine significance, while for non-normally distributed data, Mann-Whitney U test was utilized. Categorical data were tested using chi-square test. P-values of p<0.05 were considered significant. The Pearson correlation coefficient was calculated to determine correlation, and the corresponding two-sided p-values were calculated using the scipy.stats.pearsonr function. For visualization, Python 3.6 with the matplotlib and seaborn libraries was utilized. For classification, support vector machine (SVM) and random forest classifier (RF) models were built with scikit-learn, and extreme gradient boosting (XGBoost) models were constructed using the XGBoost library. All medical and microbial data were utilized for model training. For model optimization using grid search, we utilized 5-fold cross-validation and for Bayesian optimization 10-fold cross-validation. The main parameters and conditions for each model were as follows: the SVM model was optimized using grid optimization (C: 0.1, 1, 10, 100, 1000; gamma: 1, 0.1, 0.01, 0.001, 0.0001, 0.00001, 0.000001). Grid search results were ranked based on AUC scores and cross-validated five times. RF models were optimized in 30 iterations and 5-fold cross-validation using a randomized grid (estimators: 100, 307, 514, 721, 928, 1135, 1342, 1550, 1757, 1964, 2171, 2378, 2585, 2792, 3000; max depth: 1, 5, 10, 20, 50, 75, 100, 150, 200; minimum samples split: 1, 2, 5, 10, 15, 20, 30; minimum samples leaf: 1, 2, 3, 4; max features: auto/square root; bootstrap: yes/no; criterion: gini/entropy), and then optimized using grid search with 5-fold cross-validation (estimators: between 2000 and 2300 by 5; max depth: 180, 190, 200, 210, 220; minimum samples split: 2, 3, 4; minimum samples leaf: 1, 2, 3, 4; bootstrap: yes; criterion: gini). For the XGBoost model, 10-fold cross-validation with 100 iterations, using Bayesian optimization, was utilized (maximum depth: 2–8; learning rate: 0.001–1; subsample: 0.5–1; columns sampled by tree, level, and node: 0.5–1; alpha: 0–10; lambda: 0–10; gamma: 0–10).

Data preprocessing included the use of the Synthetic Minority Oversampling Technique (SMOTE) to oversample the minority class and prevent overfitting, as well as basic data standardization. Data were randomly split, with 80% used for training and 20% for testing.

SHapley Additive exPlanations (SHAP) analysis was used to determine feature importance for all three different models. It enables the comparison of multiple machine learning models by assigning interpretable and consistent feature contribution scores based on Shapley values, allowing differences in feature importance across models to be directly analyzed (Lundberg and Lee, 2017).

## Results

### Relevant clinical data in the study groups

The preliminary dataset comprised 488 couples. Microbial data were unavailable for 13 female patients, largely attributable to the use of donor sperm or cryopreserved testicular samples during IVF, and for 26 male patients. Accordingly, analyses were conducted using data from 475 women and 462 men, with paired male–female data available for 455 couples.

In the comparison of clinical and procedural characteristics between women with negative (n=354) and positive (n=121) microbiological cultures of vaginal discharge (**Table 1**), most biometric and infertility-related parameters did not differ significantly between the groups. Maternal age, body weight, height, body mass index, duration of infertility, baseline hormonal parameters (FSH, LH, AMH), as well as ovarian stimulation characteristics (duration of stimulation, number of follicles, endometrial thickness, and timing and number of embryo transfers) showed no significant variation.

**Table 1:** Relevant clinical data in the study groups.

Clinical data	Negative microbiological culture of vaginal discharge (n=354)	Positive microbiological culture of vaginal discharge (n=121)	Sig.
Biometric data			
Maternal age (years)	34.7 ± 5.07	34.5 ± 5.38	n.s.
Weight (kg)	68.8 ± 16.02	68.3 ± 14.31	n.s.
Height (cm)	165.5 ± 6.33	165.4 ± 5.89	n.s.
BMI (kg/m^2^)	25.1 ± 5.41	25.0 ± 5.04	n.s.
Preprocedural data			
Duration of infertility (years)	3.9 ± 2.79	3.5 ± 2.34	n.s.
Previous live births (n; %)	48 (14)	26 (21)	*
Previous miscarriages (n; %)	55 (16) n=337	19 (16)	n.s.
Previous induced abortions (n; %)	43 (13) n=338	15 (12)	n.s.
Fallopian tube obstruction (n; %)	89 (25)	26 (22)	n.s.
Unsuccessful IUI (n; %)	85 (24)	29 (24)	n.s.
PCOS (n; %)	21 (6)	12 (10)	n.s.
Endometriosis (n; %)	28 (8)	12(10)	n.s.
Male-factor infertility (n; %)	141 (40	50 (41)	n.s.
FSH (IU/L)	7.8 ± 3.27	8.1 ± 3.55	n.s.
LH (IU/L)	5.8 ± 2.43	6.6 ± 4.28	n.s.
AMH (pmol/L)	3.9 ± 4.49	3.1 ± 3.72	n.s.
Lactobacillus cultured (n; %)	232 (78) (n=296)	36 (35) (n=104)	n.s.
Microscopic image of bacterial vaginosis (n; %)	25 (8) (n=296)	22 (21) (n=107)	n.s.
Granulocyte observed under microscopy (n; %)	43 (14) (n=297)	7 (7) (n=104)	n.s.
Procedural data			
Duration of stimulation (days)	10.2 ± 1.84	10.39 ± 1.92	n.s.
Number of follicles	8.72 ± 5.40	8.6 ± 4.65	n.s.
Day of embryo transfer	4.3 ± 0.78	4.3 ± 0.88	n.s.
Number of embryos transferred per patient	1.6 ± 0.53	1.6 ± 0.53	n.s.
Implantation rate	0.2 ± 0.36	0.3 ± 0.40	n.s.
ENDOV (mm)	10.1 ± 2.05 (n=348)	10.3 ± 1.87 (n=121)	n.s.
ENDPU (mm)	10.6 ± 2.04 (n=320)	10.8 ± 1.95 (n=115)	n.s.
ENDET (mm)	11.7 ± 2.60 (n=350)	11.3 ± 2.37 (n=119)	n.s.
Paternal side parameters			
Paternal age (years)	37.8 ± 6.30	36.8 ± 6.07	n.s.
Positive microbiological culture of semen (n; %)	97 (29)	47 (41)	n.s.
Positive microbiological culture of semen weighted	0.3 ± 0.47	0.4 ± 0.64	**
Sperm concentration (x 10^6^/ml)	50.7 ± 39.85	49.8 ± 38.82	n.s.
Sperm motility (%)	44.6 ± 17.95	43.8 ± 17.33	n.s.
Normal sperm morphology > 4% (n; %)	235 (70)	84 (75)	n.s.
Outcome			
Pregnant patients (n; %)	112 (36)	44 (39)	n.s.

The data are presented as mean ± SD or number and percentage (n,%); n.s., not significant; *p-value<0.05; **p<0.01. The clinical data were grouped to subcategories marked with bold letters. FSH, follicle-stimulating hormone; LH, luteinizing hormone; AMH, anti-Müllerian hormone; ENDOV, endometrial thickness on the day of triggering human chorionic gonadotropin injection; ENDPU, endometrial thickness on the day of puncture; ENDET, endometrial thickness at the time of embryo transfer; IUI, intrauterine insemination; PCOS, polycystic ovary syndrome; Sig., significance.

Among the preprocedural reproductive history, the proportion of women with previous live births was significantly higher in the group with positive vaginal cultures compared to those with negative cultures (21% vs. 14%, p<0.05). No differences were found in the rates of previous miscarriages or induced abortions. Microscopic and culture-based findings regarding vaginal flora, including the presence of *Lactobacillus*, bacterial vaginosis, or granulocytes, were not significantly different between groups.

On the paternal side, no significant differences were observed in paternal age, sperm concentration, motility, or morphology. However, a positive microbiological semen culture was considerably more frequent in partners of women with positive vaginal cultures (41%) compared to the negative group (29%).

Finally, clinical pregnancy rates did not differ significantly, with rates of 39% in the positive vaginal culture group compared with 36% in the negative group.

### Results of the vaginal and semen microbiota analysis

**Figure 1:**
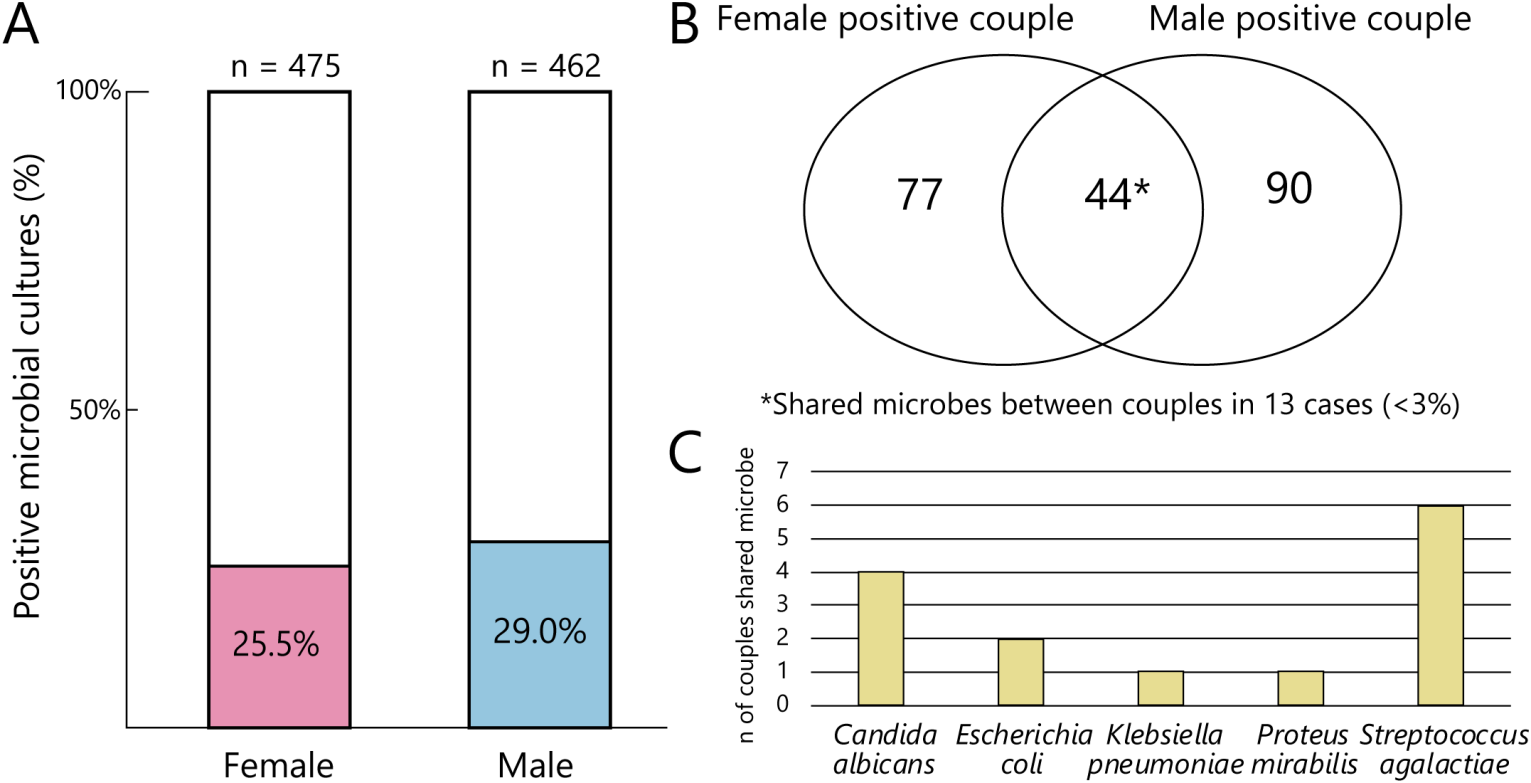
Frequency and composition of microbial co-occurrence in the examined samples. **(A)** Bar chart represents the percentage of positive microbial tests of the whole dataset between sexes. **(B)** Venn diagram depicts the distribution of female and male positive microbial tests of the couples. **(C)** Bar chart represents the occurrence of microbes that are shared between the couples (n_couples_=13).

In total 121 female patients had detectable microbial infection, with 148 detected microbes, meanwhile 136 male patients had positive samples and 147 microbes were detected. In most cases, only a single microbe was detectable in both women’s (82%) and men’s (93%) samples (**Figure 1**). We investigated the occurrence of microbes in two ways: using a binary categorization and a weighted occurrence, where we summed the number of different microbes present.

In cases where multiple microbes were detected, this include 23 cases of female samples showing the presence of 2 microbes, while among male samples, 9 had 2 microbes and 1 had 3 microbes. The dual occurrence of pathogens involved 14 species in female samples and 9 species in male samples. The sample size does not allow us to determine a considerable co-occurrence of the microbes; the observed occurrences seem to be random. The frequency and composition of microbial co-occurrence in the examined samples is presented in **Figure 1**.

**Figure 2:**
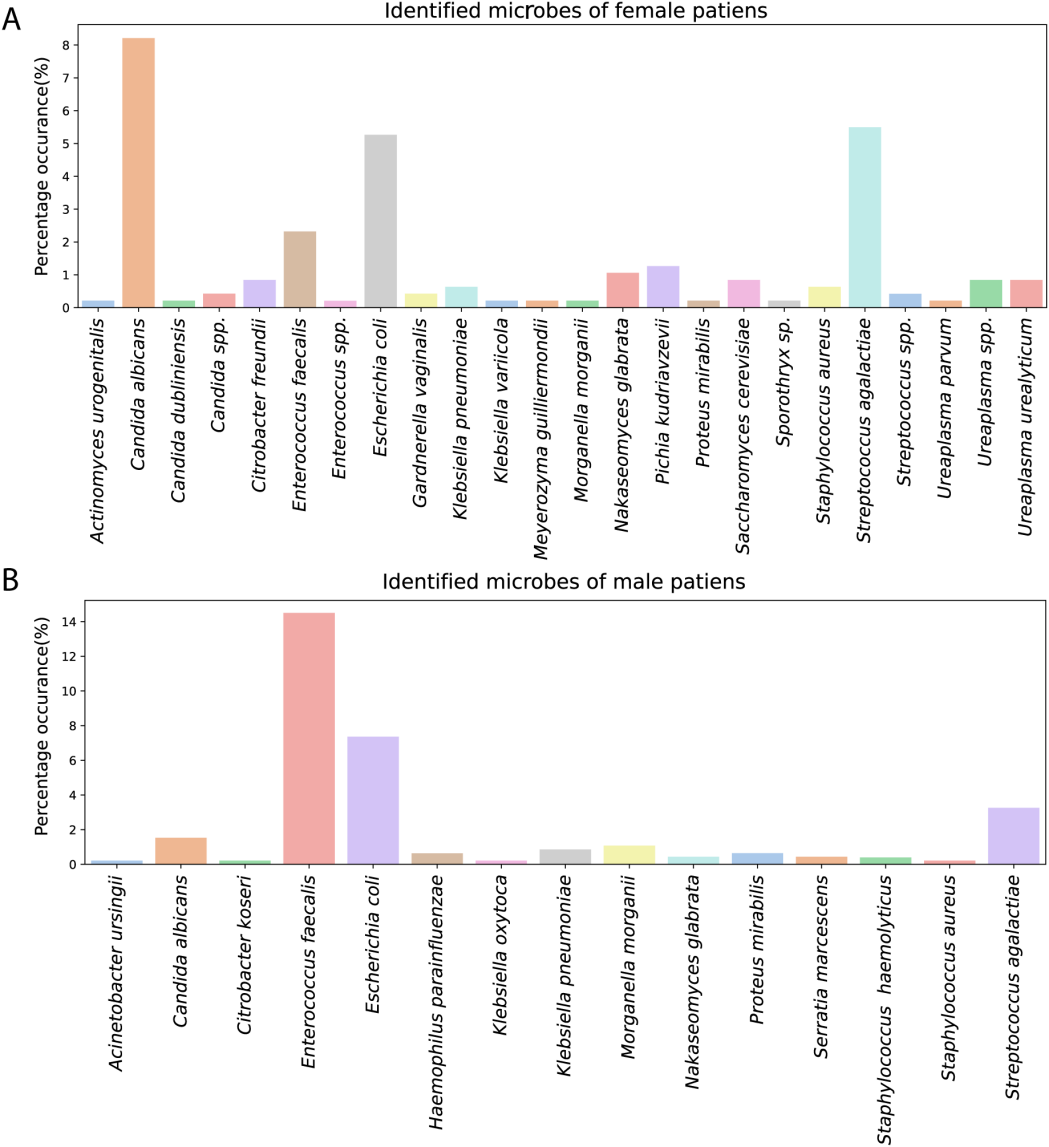
**(A)** Results of the positive vaginal discharge microbiota analysis: distribution of pathogens (n=475). **(B)** Results of the positive semen microbiota analysis: distribution of pathogens (n=462). Percentages represent the occurrence in the whole dataset.

Regarding the total population, only a low number of isolates yielded positive results, as most pathogens occurred in less than 5% of the isolates (**Figure 2**). In isolates obtained from female patients, the top three pathogens were *Candida albicans* at 8%, *Streptococcus agalactiae* at 5%, and *Escherichia coli* at 5%. In isolates from the male patients, *Enterococcus faecalis* (14%) and *Escherichia coli* (7%) were the dominant isolates.

**Figure 3:**
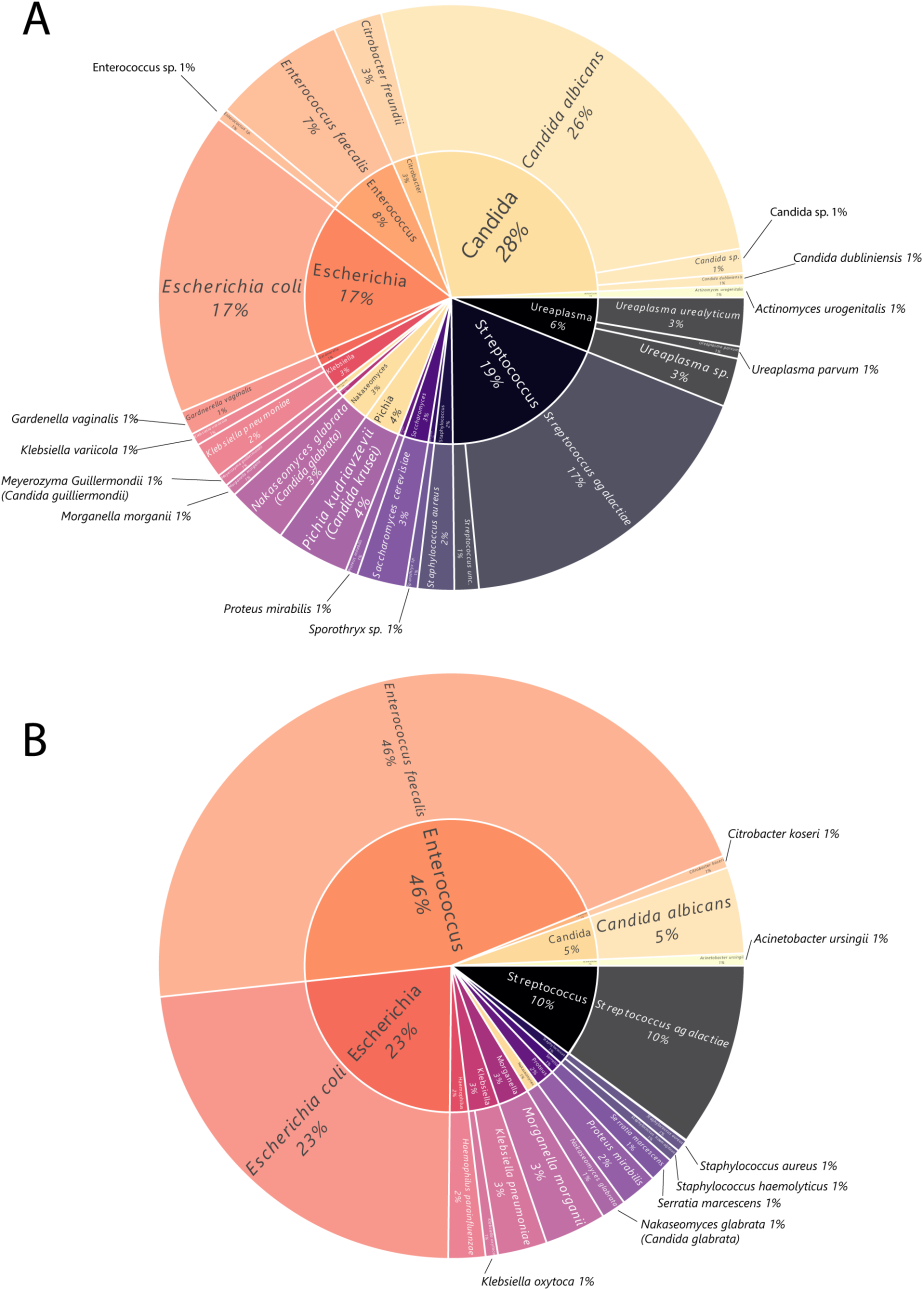
**(A)** Pie chart illustrating the distribution of pathogens identified in positive vaginal secretion samples (n_microbes identified_ = 148). **(B)** Pie chart illustrating the distribution of pathogens identified in positive semen samples (n_microbes identified_ = 147). Percentages represent the distribution among positive samples.

**Figure 2A** presents the percentage of pathogens identified in 121 positive vaginal microbiological cultures, while **Figure 3A** depicts the proportional distribution of the identified microbes. In the female cohort, *Candida albicans* represented the most frequently identified microorganism, accounting for 26% of all positive cases among women. This microorganism was more prevalent among non-pregnant women, who exhibited a higher occurrence (26%) compared to pregnant women (22%). Other common isolates in the female group included *Streptococcus agalactiae* (17%) and *Escherichia coli* (17%), with *Enterococcus faecalis* contributing to 7% of positive cases. *Pichia kudriavzevii* (formerly *Candida krusei*) and *Nakaseomyces glabrata* (previously known as *Candida glabrata)* were less frequently detected, accounting for 4% and 3%, respectively. Additionally, a wide range of less prevalent microorganisms, such as *Gardnerella vaginalis, Actinomyces urogenitalis, Ureaplasma species*, and several rare yeasts (e.g., *Saccharomyces cerevisiae, Meyerozyma guilliermondii* -formerly *Candida guilliermondii*- and *Candida dubliniensis*), with their occurrence being sporadic. Overall, the distribution pattern suggested that pathogenic and opportunistic species were more common in the non-pregnant subgroup (Mändar et al., 2015), while the pregnant cohort displayed a narrower microbial spectrum (Sakamoto, 1985).

**Figure 2B** presents the percentage of pathogens identified in 136 positive semen microbiological cultures, while **Figure 3B** depicts the proportional distribution of the identified microbes. In the male population, *Enterococcus faecalis* was the dominant microorganism, detected in 46% of positive cases among men. This was followed by *Escherichia coli*, which made up 23% of the cases, and *Streptococcus agalactiae*, which accounted for 10%. *Candida albicans* was detected in 5%, while *Klebsiella pneumoniae* (3%) and *Morganella morganii* (3%) were observed in lower frequencies. Less common bacteria, including *Proteus mirabilis, Haemophilus parainfluenzae, Serratia marcescens, and various Staphylococcus species*, were found in small amounts. Rare isolates, such as *Klebsiella oxytoca, Citrobacter freundii*, and *Acinetobacter ursingii*, were only sporadically present.

The male cohort demonstrated a more diverse range of microorganisms, with *Enterococcus faecalis* and *Escherichia coli* remaining the predominant pathogens, especially in men with non-pregnant partner (47% and 22%, respectively).

Taken together, these findings highlight that while both the vaginal and seminal microbiota exhibited a diverse microbial composition, a limited number of pathogens (*Candida albicans, Enterococcus faecalis, Escherichia coli, Streptococcus agalactiae*) accounted for the majority of positive cultures.

**Figure 4:**
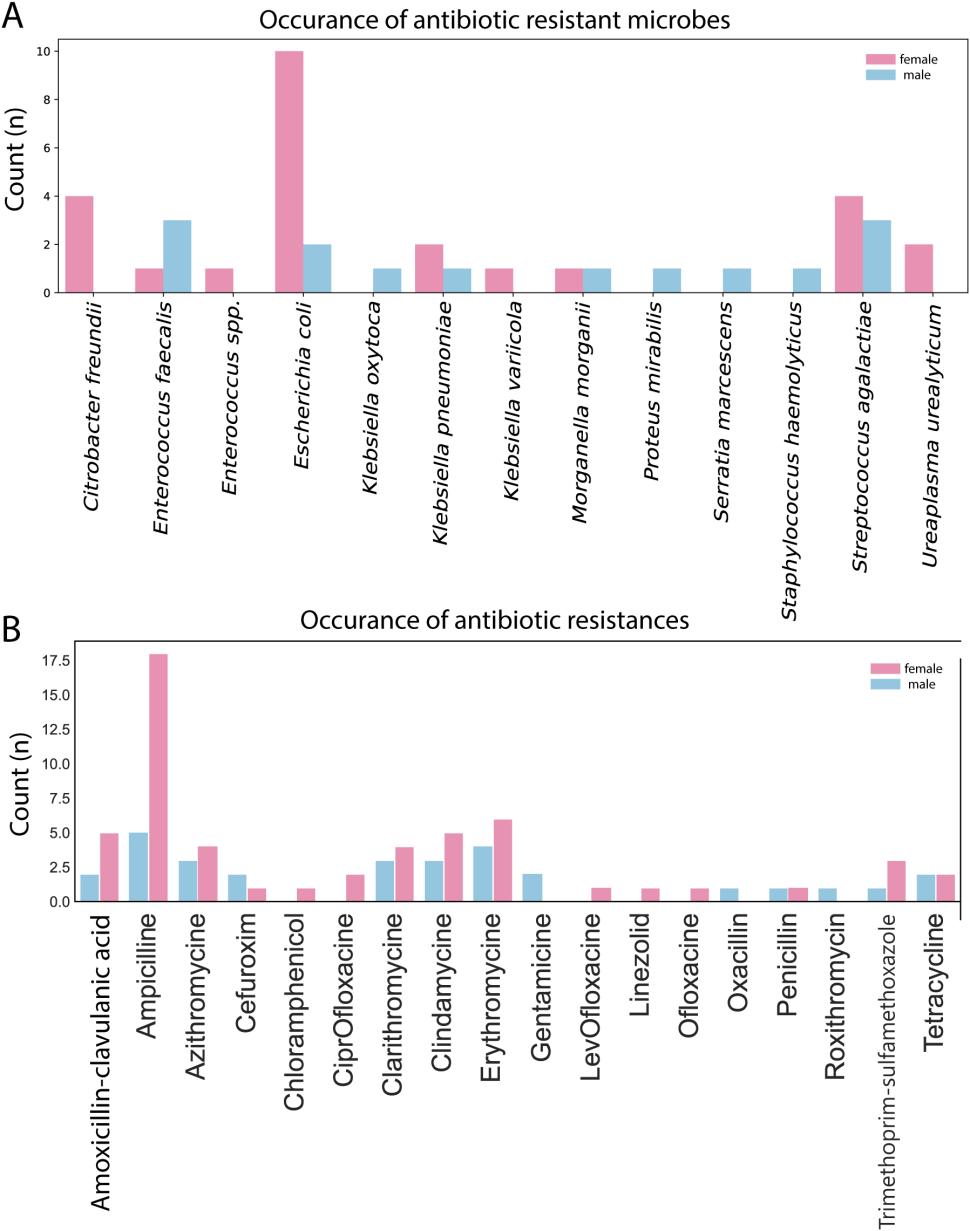
**(A)** The distribution of antimicrobial resistance of microbes between male and female samples. **(B)** The resistance types with occurrences distribute between male and female patients.

In 110 cases the antimicrobial resistance was also tested. Antimicrobiotic resistant strains occur in a similar pattern as the general flora, in female side tests resistances occur mostly related to *Escherichia coli*, *Citrobacter freundii* and *Streptococcus agalactiae*. On the male side most prominent microbes with resistance were *Enterococcus feacalis*, *Streptococcus agalactiae* and *Escherichia coli* (**Figure 4A**). We detected resistance against 16 antibiotics. Female samples showed considerably more resistance than male samples most notably ampicillin resistance was very prominent (**Figure 4B**).

The effect of the presence of antibiotic resistant microbe strains did not influence the pregnancy (non-pregnant: 49 [15%], pregnant: 27 [17%]) neither in female (non-pregnant: 21 [6%] pregnant: 13 [8%]) or male patients (not pregnant: 28 [9%], pregnant: 14 [9%]).

### *Lactobacillus* presence versus absence

In the comparison of women with vaginal *Lactobacillus* colonization (n=270) vs. those without (n=132), age distribution did not differ significantly between the two groups (34.7 ± 5.3 vs. 34.8 ± 5.1 years, n.s.). The prevalence of bacterial positivity in vaginal cultures was significantly higher in the Lactobacillus-negative group compared with those with detectable *Lactobacillus* (13% [36/268] vs. 52% [68/132], p < 0.001). The occurrence of Candida species was somewhat higher in the Lactobacillus-negative group (18% [24/132]) compared with the Lactobacillus-positive group (8% [22/270]), but this difference was not significant. No other microorganisms exhibited consistent or statistically significant differences between the groups. Furthermore, when comparing these two groups, no additional reliable significant differences were observed in preprocedural or procedural characteristics, and clinical pregnancy rates were comparable between the groups.

### Correlations

Vaginal discharge microbiological positivity correlated weakly but significantly with semen bacterial positivity (r=0.13, p=0.007) and also with the number of previous deliveries (r=0.09, p=0.049).

The presence of *Lactobacillus* in vaginal discharge cultures showed significant negative correlations with several pathogens, including *Candida albicans* (r=−0.13, p=0.008), *Streptococcus agalactiae* (r=−0.15, p=0.003), *Gardnerella vaginalis* (r=−0.14, p=0.033), *Enterococcus faecalis* (r=−0.23, p<0.001), *Escherichia coli* (r=−0.31, p<0.001), *Staphylococcus aureus* (r=−0.12, p=0.013), *Klebsiella pneumoniae* (r=−0.12, p=0.013), and with bacterial vaginosis diagnosed microscopically (r=−0.45, p<0.001).

Bacterial vaginosis was positively correlated with the presence of *Actinomyces urogenitalis* (r=0.12, p=0.012), *Streptococcus agalactiae* (r=0.10, p=0.038), *Gardnerella vaginalis* (r=0.23, p<0.001), and *Candida guilliermondii* (r=0.11, p=0.022). In addition, bacterial vaginosis also showed a weak but significant positive correlation with serum AMH levels (r=0.10, p=0.041) and a negative correlation with the duration of infertility (r=−0.14, p=0.005). The full list of correlation coefficients is available in the **Supplementary Material**, **Supplementary Table 1**.

### Contribution of microbiological data to machine learning model performance

**Figure 5:**
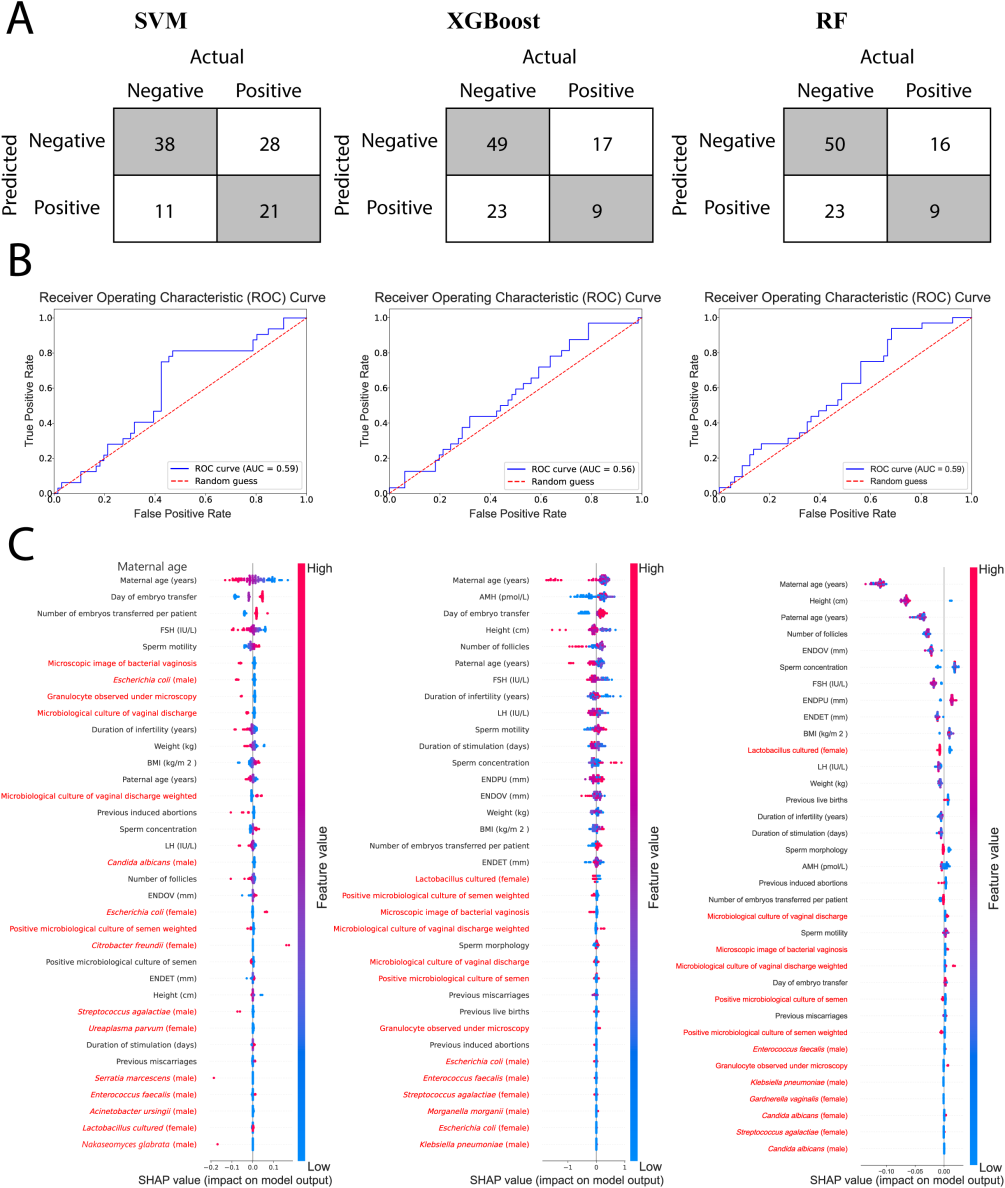
Machine learning assessment of the data. SVM results are represented in the first column, XGBoost in the second, and RF in the third column. **(A)** Tables represent the confusion matrices of the models. **(B)** Graphs represent the ROC/AUC curves of the models. **(C)** Graphs depict the SHAP values of the first 35 features of each model. Microbiological data-associated feature names are highlighted in red.

To assess the contribution of microbial infection to fertility outcomes, we used machine learning algorithms. We utilized all available patient data with 70 dimensions, including medical and microbial data, to train the models. Out of the 70 categories, 48 (68.6%) were derived from the microbiological data (**Supplementary Material**, **Supplementary Table 1**). Support vector machine (SVM), random forest classifier (RF), and extreme gradient boosting (XGBoost) were utilized. The models performed poorly in general (**Figures 5A, B**), with an AUC score of no more than 0.59. All three models showed a similar accuracy (SVM: 60.2%, XGBoost: 59.2%, RF: 60.2%); however, the SVM model was better at predicting positive outcomes (positive predictive value – SVM: 65.6%, XGBoost, RF: 28.1%). Meanwhile, the two decision tree-based methods were better at negative predictions (negative predictive value - SVM: 57.6%, XGBoost: 74.2%, RF: 75.8%). Interestingly, the SVM model had the highest specificity (77.6%) and sensitivity (42.9%) compared to XGBoost (68.1%, 34.6%) and RF (68.5%, 36.0%). These results show that the current amount of data is insufficient to achieve higher performance. Despite the poor prospects of using these models for prediction, the possible contribution of each dimension is still intriguing. We used SHAP analysis to determine the most important features of the models. SHAP is advantageous for comparing machine learning models because it provides a consistent explanation of predictions for each model, making it possible to directly evaluate how strongly each model relies on the input features. The first 35 features were investigated for each model (**Figure 5C**). As expected, maternal age was a fundamental predictor in all three models. The SVM model’s top features included 19 clinical data points and 16 microbial data points, with the most important predictors belonging to clinical data. The decision tree-based methods both showed similar patterns, where microbial data contributed poorly to the models. In both models, the first microbial feature was the presence of lactobacillus cultures. In conclusion, microbial data contributed poorly to the ML models. Some niche factors might be identified with SVM, yet overall, lactobacillus culture appears to be the most promising factor.

## Discussion

Comparison of clinical and procedural characteristics between women with negative and positive microbiological cultures of vaginal discharge (**Table 1**) revealed no significant differences in most biometric, preprocedural, or procedural parameters, nor in microscopic and culture-based assessments of vaginal flora, including the presence of *Lactobacillus*, bacterial vaginosis, or granulocytes. Only the proportion of women with previous live births was significantly higher in the group with positive vaginal cultures compared to those with negative cultures (21% vs. 14%, p<0.05). To the best of our knowledge, this association is described here for the first time.

Our findings indicate that semen culture positivity is more prevalent among male partners of women with positive vaginal cultures. Nevertheless, in contrast to the observations of Ricci et al (Ricci et al., 2018), we did not detect a significant association with pregnancy outcomes. This suggests that, in our cohort, microbial colonization—although demonstrable in both partners—may not exert a clinically meaningful impact on implantation or early pregnancy.

In female samples, the most prevalent pathogens were *Candida albicans* (8%), *Streptococcus agalactiae* (5%), and *Escherichia coli* (5%), whereas in male samples, *Enterococcus faecalis* (14%) and *Escherichia coli* (7%) were the dominant isolates. Similarly, in the study by Ricci et al (Ricci et al., 2018), *Enterococcus faecalis* and *Streptococcus agalactiae* were the most frequently detected microbes in males and females, respectively. The male cohort exhibited a greater microbial diversity, a finding consistent with the observations reported by Okwelogu et al (Okwelogu et al., 2021).

Identical microbial species were detected in only 13 couples during microbiological culture (**Figure 1**), which can be considered a rather unexpected finding. To the best of our knowledge, no similar investigation has been conducted previously in Hungary; this is the first study to demonstrate that pre-IVF microbiological culture identifies the presence of the same pathogen in fewer than 3% of couples. The prospective study of Ricci et al. (2018) investigated the prevalence of asymptomatic genital tract infections among 285 infertile couples undergoing IVF. At least one genital pathogen was isolated in 46.3% of couples. Both partners tested positive in 26 couples, and in 16 of these, the same pathogen was identified in both partners (≈5.6% of all couples) (Ricci et al., 2018).

Several factors may contribute to the limited microbial concordance observed between partners. Microbiological sampling was performed at a single pre-treatment time point, while genital tract microbiota are known to exhibit temporal variability. The vaginal and seminal microbial communities differ substantially in their ecological environments (Baud et al., 2023), which may limit stable cross-colonization. Behavioral factors such as variability in sexual activity frequency (Mändar et al., 2015) or the interval between intercourse and sampling may also influence the probability of detecting shared microorganisms. Microbial transmission during sexual intercourse appears to occur largely through stochastic processes rather than stable deterministic colonization (Ma, 2022).

According to a recent review (Molina et al., 2025) which introduced the concept of the “seminovaginal microbiome”, highlighting the dynamic interaction between the seminal and vaginal microbial communities. Although it does not provide precise prevalence data, it emphasizes that microbial overlap between partners is biologically plausible and clinically relevant in the context of assisted reproduction.

Furthermore, in our study, the distribution pattern indicated that pathogenic and opportunistic species were more prevalent in the non-pregnant subgroup, whereas the pregnant cohort exhibited a more restricted microbial spectrum. Previous study (Bar et al., 2025) observed that higher microbial diversity is inversely associated with the likelihood of achieving a clinical pregnancy. Freitas et al. (2017) observed that the vaginal microbiome of healthy pregnant women was characterized by reduced richness and diversity compared to non-pregnant women.

A *Lactobacillus*-dominated microbiome profile has been positively linked to favorable reproductive outcomes (Koedooder et al., 2019; Ji et al., 2022), whereas the presence of bacterial vaginosis has been associated with diminished success rates (Schoenmakers and Laven, 2020). Recent ART innovations allow clinicians to monitor vaginal microbiome profiles to optimize the timing of IVF and embryo transfer. Predictive tests can estimate pregnancy likelihood based on vaginal microbiome composition, assigning high, medium, or low probability scores according to *Lactobacillus* abundance, *Lactobacillus jensenii* proportion, presence of *Gardnerella vaginalis* IST1, and relative *Proteobacteria* load (Haahr et al., 2016).

In our study, as expected, the presence of *Lactobacillus* spp. in vaginal discharge cultures demonstrated an inverse association with multiple pathogens, including *Candida albicans*, *Streptococcus agalactiae, Gardnerella vaginalis, Enterococcus faecalis, Escherichia coli, Staphylococcus aureus*, and *Klebsiella pneumonia*, as well as with microscopically diagnosed bacterial vaginosis. Our results are consistent with the study of Ricci et al (Ricci et al., 2018), who also found in their analysis of female genital swabs that the presence of *Enterococcus faecalis*, *Escherichia coli, Streptococcus agalactiae*, and *Gardnerella vaginalis* was significantly associated to reduced levels of vaginal *Lactobacilli*. Recent evidence suggests that a *Lactobacillus*−dominant vaginal microbiome promotes IVF success by maintaining low vaginal pH (Savicheva et al., 2023; Wang et al., 2024), suppressing pathogenic overgrowth (Miko and Barakonyi, 2023), competitively inhibiting microbial adhesion (He et al., 2020), and modulating local immune responses (Avitabile et al., 2024), collectively creating a reproductive environment favorable for implantation and early pregnancy.

In our study, bacterial vaginosis was positively associated with the presence of *Actinomyces urogenitalis*, *Streptococcus agalactiae, Gardnerella vaginalis*, and *Meyerozyma guilliermondii*, consistent with previous reports linking these microorganisms to dysbiotic vaginal environments (Nikolaitchouk et al., 2008; Cocomazzi et al., 2023). It is noteworthy that the observed correlations are relatively weak and, in most cases, cannot be considered strong associations.

In this study, we applied multiple machine learning algorithms to investigate whether microbial infection data could enhance the prediction of fertility outcomes. Despite extensive model optimization and the inclusion of 70 dimensions, nearly 70% of which were derived from microbiological data, our models performed poorly (AUC ≤ 0.59). These findings indicate that the current dataset is insufficient to achieve robust predictive performance, which is consistent with previous reports emphasizing the multifactorial and heterogeneous nature of fertility prediction. The SHAP analysis confirmed maternal age as the dominant predictor across all models, in line with established evidence that age remains the most important factor influencing reproductive success (Peng et al., 2024; Nádasdi et al., 2025). The overall contribution of microbial features was modest. This limited predictive value of microbiome-related parameters may stem from the relatively small sample size, the variability in microbiome profiling methods, or the secondary role of microbial infections compared to major clinical predictors.

Although reporting antimicrobial resistance patterns represents a unique strength of our study, the current analysis did not explore a relationship between these resistance profiles and clinical pregnancy outcomes. Prior research suggests that certain resistant bacterial strains in the reproductive tract may influence sperm quality or endometrial receptivity, potentially affecting fertility outcomes (Shash et al., 2023; Wang et al., 2024). Integrating resistance data into predictive models could provide additional insights into the clinical relevance of microbial colonization beyond mere presence or absence. Future studies should consider evaluating whether specific resistant pathogens, alone or in combination with microbiome composition, modulate IVF success rates.

Nevertheless, our models consistently identified *Lactobacillus* as the leading microbial factor, which aligns with growing evidence that a *Lactobacillus*-dominant genital tract microbiome is beneficial for IVF outcomes. Guan et al. (2023) demonstrated that *Lactobacillus crispatus* dominance in the cervical microbiome was associated with higher implantation and clinical pregnancy rates in frozen embryo transfer cycles. Similarly, Alley et al. (2025) reported improved reproductive outcomes in women with *Lactobacillus*-dominant vaginal microbiota.

Taken together, our findings suggest that while microbial infection data alone may not provide sufficient predictive power for fertility outcomes in machine learning models, the consistent identification of *Lactobacillus* supports its potential as a clinically relevant biomarker. Future studies with larger, multi-center cohorts and standardized microbiome profiling are warranted to validate these associations and better integrate microbial signatures with established clinical predictors.

## Strengths and limitations

A limitation of our study is that the results of microbiological culture and microscopic analysis were obtained from different diagnostic centers located in multiple cities across three countries (Hungary, Romania, and Serbia). Consequently, the methodology was not fully standardized, which may have introduced variability into the findings. However, this heterogeneity also reflects real-world clinical practice and thus enhances the validity of our results.

A limitation of our study is that the microbiological analysis was based on culture-dependent methods, which capture only a subset of the vaginal microbial community. Conventional microbiologic cultivation and next-generation sequencing (NGS) approaches, particularly 16S rRNA gene profiling, provide fundamentally different yet complementary perspectives on the vaginal microbiome. Culture-based methods selectively detect viable and metabolically active microorganisms, thereby offering clinically relevant insights into microbial function and host interaction. In contrast, 16S rRNA sequencing identifies bacterial DNA irrespective of viability, capturing signals from live, dormant, and dead organisms, as well as extracellular genetic material. This distinction may lead to discrepancies in microbial composition, with NGS often reporting greater diversity than culture-based techniques (Sola-Leyva et al., 2026). Such differences raise important questions regarding the biological and clinical relevance of detected taxa, particularly in low-biomass or dynamic environments such as the vaginal niche. While NGS expands detection capacity beyond cultivable species, it may overestimate the functional microbiota by including non-viable contributors. Conversely, culture-based approaches may underestimate diversity due to methodological constraints and selective growth conditions. Emerging methodologies, including viability polymerase chain reaction (PCR) and transcriptomic analyses, aim to bridge the gap by integrating compositional and functional data. A comprehensive understanding of the vaginal microbiome therefore requires careful interpretation of both viability and genetic presence. Integrative approaches combining culture and molecular techniques are likely to provide the most accurate representation of microbial ecology and its clinical implications. Nevertheless, culture-based diagnostics retain important clinical advantages, as they enable the isolation of viable pathogens and allow antibiotic susceptibility testing, which is essential for guiding appropriate antimicrobial therapy. Future studies combining culture-based methods with NGS approaches may provide a more complete understanding of the vaginal microbiome and its potential impact on reproductive outcomes.

Another limitation of this study is its retrospective design, which may introduce selection bias and incomplete data availability. In addition, microbiological sampling was performed at a single pre-treatment time point; therefore, temporal variability of the genital microbiota could not be assessed. However, the primary objective of the present study was not to investigate longitudinal microbiome dynamics but to evaluate associations between baseline microbiological culture results and IVF outcomes in a large clinical cohort.

## Conclusion

The main findings of our study are: (i) vaginal and semen culture positivity is correlated between partners, (ii) prior live births may be more frequent among women with positive vaginal cultures, (iii) the presence of positive vaginal cultures was not significantly associated with IVF negative outcome, (iv) our study is the first in Hungary to demonstrate that pre-IVF microbiological culture identifies the presence of the same pathogen in fewer than 3% of couples, (v) pathogenic and opportunistic species were more common in the non-pregnant subgroup, while the pregnant cohort displayed a narrower microbial spectrum, (vi) the presence of *Lactobacillus* in vaginal discharge cultures showed significant negative correlations with *Candida albicans, Streptococcus agalactiae, Gardnerella vaginalis, Enterococcus faecalis, Escherichia coli, Staphylococcus aureus, Klebsiella pneumoniae*, and bacterial vaginosis, (vii) while microbial infection data alone may not provide sufficient predictive power for fertility outcomes in machine learning models, the consistent identification of *Lactobacillus* supports its potential as a clinically relevant biomarker.

In summary, our data suggest that while vaginal and semen culture positivity are correlated between partners and prior live births may be more frequent among women with positive vaginal cultures, culture positivity per se does not appear to correlate with impaired IVF clinical pregnancy rates in our cohort. These findings should be interpreted cautiously, as the present analysis is exploratory and based on retrospective data. The results suggest that not all microbial colonization is clinically consequential, and that species‐level characteristics, microbial load, and community composition may modulate potential effects. Further prospective studies with standardized microbiome assessment are required before clinical implications can be established. Future research integrating conventional culture-based diagnostics with genomic microbiome profiling (e.g., 16S rRNA sequencing or metagenomic approaches) (Sola-Leyva et al., 2026) may improve the predictive performance of microbiological data in fertility outcome models. Ultimately, this research endeavors to contribute to a more nuanced understanding of infertility’s multifactorial etiology and support the advancement of personalized fertility management strategies.

## Data Availability

The original contributions presented in the study are included in the article/**Supplementary Material**. Further inquiries can be directed to the corresponding author.

## Glossary

IVF: *in vitro* fertilization
WHO: World Health Organization
ART: assisted reproductive technology
SVM: support vector machine
RF: random forest classifier
XGBoost: extreme gradient boosting
AMH: Anti-Müllerian hormone
LH: luteinizing hormone
