# Supplementary material for: Retrospective comparative analysis of pre-IVF vaginal and semen microbiological cultures in infertile couples highlights absence of microbial concordance
Source: Front Cell Infect Microbiol. 2026 Apr 29;16:1747088. doi: 10.3389/fcimb.2026.1747088 (PMC13168007; doi:10.3389/fcimb.2026.1747088)
Supplement: Supplementary file 1 [file Supplementaryfile1.docx]

**Retrospective Comparative Analysis of Pre-IVF Vaginal and Semen Microbiological Cultures in Infertile Couples Highlights Absence of Microbial Concordance**

**Supplementary Material**

**Methods**

Considering each woman’s ovarian function, hormonal profile, age, and body weight, personalized ovarian stimulation regimens were applied. The majority of patients received a flexible GnRH-antagonist protocol, while GnRH-agonist stimulation was used less frequently. Cycle initiation was scheduled for the 2nd or 3rd day of menstruation. During the first visit, patients underwent transvaginal ultrasonography (Samsung Medison HS50; endovaginal probe: EVN4-9, 4–9 MHz), evaluation of the antral follicle count, and measurement of serum FSH, LH, prolactin, and thyroid-stimulating hormone levels. Ovarian stimulation with FSH was started on cycle day 2–3, and the dose was subsequently modified based on ovarian response, monitored with serial ultrasound and serum estradiol measurements beginning on day 5 and then every 2–3 days. Once the leading follicles reached 14 mm, daily administration of 0.25 mg GnRH antagonist was commenced and continued until ovulation triggering. When at least three follicles measured ≥18 mm, ovulation was induced with hCG. Oocyte retrieval took place 36 h after hCG injection (Ovitrelle®, Merck KGaA, Merck Serono, Darmstadt, Germany).

Laboratory procedures and embryo transfer (ET) were performed in accordance with standardized protocols across all cycles. Follicular fluid was collected in preheated 14 mL round-bottom tubes (Thermo Fisher Scientific Inc., Roskilde, Denmark) and maintained at 37 °C in a heating block. Oocyte identification was carried out under laminar flow using 90 mm petri dishes (Thermo Fisher Scientific Inc., Roskilde, Denmark). The cumulus–oocyte complexes were transferred into Nunc IVF Center Well Dishes (Thermo Fisher Scientific Inc., Roskilde, Denmark) containing G-MOPS PLUS medium (Vitrolife AB, Gothenburg, Sweden), followed by transfer into 5-well dishes (Vitrolife AB, Gothenburg, Sweden) with G-IVF medium (Vitrolife AB, Gothenburg, Sweden) covered by Hypure Heavy Ovoil (Kitazato Corp., Shizuoka, Japan). Incubation was performed under tightly controlled conditions of 37 °C, 6% CO2, and 5% O2 (K-Systems G210 InviCell, CooperSurgical, Birkerød, Denmark, and PLANER Benchtop Incubator BT37, CooperSurgical Inc., Birkerød, Denmark).

Fertilization was achieved 2–4 h after oocyte retrieval either by conventional IVF or by ICSI, depending primarily on semen characteristics. In cases of conventional IVF, only fresh semen samples showing normozoospermia were used, and cumulus–oocyte complexes were cultured in 5-well dishes with G-IVF medium under oil. For ICSI, oocytes were denuded with hyaluronidase (80 IU/mL; SynVitro Hyadase, Origio, Ballerup, Denmark) prior to injection. Following insemination, zygotes were cultured in G1 and G2 media (Vitrolife AB, Gothenburg, Sweden) in 5-well dishes under conditions of 37 °C, 6% CO2, and 5% O2 (K-Systems G210 InviCell, CooperSurgical Inc., Birkerød, Denmark; PLANER Benchtop Incubator BT37, CooperSurgical Inc., Birkerød, Denmark).

Between 17 and 20 hours post-insemination, fertilization was confirmed by identifying two pronuclei representing maternal and paternal genetic material. Zygotes with more than two pronuclei were considered abnormal and excluded. The best-quality embryos at cleavage or blastocyst stage were chosen for transfer. Only blastocysts were cryopreserved, on day 5 or 6 of culture. Embryo quality was graded based on blastomere number and degree of fragmentation (1). Prior to transfer, blastocysts were incubated in EmbryoGlue (Vitrolife AB, Gothenburg, Sweden) for 20–40 min. Embryo transfer was performed with the Wallace Embryo Replacement Catheter (soft, 18 cm, Cooper Surgical Inc., Trumbull, Connecticut, USA), under two-dimensional transvaginal ultrasound guidance (Samsung Medison HS50; Samsung Electronics Co., Ltd./Samsung Medison Co., Ltd., endocavitary probe: EVN4-9, 4–9 MHz), which was also applied for follicular puncture. On the day of embryo transfer, endometrial thickness (mm) was measured using the same ultrasound device, from the outer edge of the endometrial–myometrial border to the opposite side at the widest point of the endometrium (2).

1. Baczkowski, T.; Kurzawa, R.; Głabowski, W. Methods of embryo scoring in in vitro fertilization. Reprod. Biol. 2004, 4, 5–22.

2. Sakamoto, C. Sonographic criteria of phasic changes in human endometrial tissue. Int. J. Gynaecol. Obstet. 1985

**Supplementary Table S1: The Excel file includes correlation data between features in the first datasheet, and a list of features used for machine learning analysis in the second datasheet**
